# A profile of Injury in Fiji: findings from a population-based injury surveillance system (TRIP-10)

**DOI:** 10.1186/1471-2458-12-1074

**Published:** 2012-12-12

**Authors:** Iris Wainiqolo, Berlin Kafoa, Bridget Kool, Josephine Herman, Eddie McCaig, Shanthi Ameratunga

**Affiliations:** 1 College of Medicine, Nursing & Health Sciences, Fiji National University, Suva, Fiji; 2 Section of Epidemiology and Biostatistics, School of Population health, University of Auckland, Private Bag, Auckland, 92019, New Zealand

**Keywords:** Injuries, Epidemiology, Injury surveillance, Fiji

## Abstract

**Background:**

Over 90% of injury deaths occur in low-and middle-income countries. However, the epidemiological profile of injuries in Pacific Islands has received little attention. We used a population-based-trauma registry to investigate the characteristics of all injuries in Viti Levu, Fiji.

**Method:**

The Fiji Injury Surveillance in Hospitals (FISH) database prospectively collected data on all injury-related deaths and primary admissions to hospital (≥12 hours stay) in Viti Levu during 12 months commencing October 2005.

**Results:**

The 2167 injury-related deaths and hospitalisations corresponded to an annual incidence rate of 333 per 100,000, with males accounting for twice as many cases as females. Almost 80% of injuries involved people aged less than 45 years, and 74% were deemed unintentional. There were 244 fatalities (71% died before admission) and 1994 hospitalisations corresponding to crude annual rates of 37.5 per 100,000 and 306 per 100,000 respectively. The leading cause of fatal injury was road traffic injury (29%) and the equivalent for injury admissions was falls (30%). The commonest type of injury resulting in death and admission to hospital was asphyxia and fractures respectively. Alcohol use was documented as a contributing factor in 13% of deaths and 12% of admissions. In general, indigenous Fijians had higher rates of injury admission, especially for interpersonal violence, while those of Indian ethnicity had higher rates of fatality, especially from suicide.

**Conclusions:**

Injury is an important public health problem that disproportionately affects young males in Fiji, with a high proportion of deaths prior to hospital presentation. This study highlights key areas requiring priority attention to reduce the burden of potentially life-threatening injuries in Fiji.

## Background

Injuries are a neglected public health problem in developing countries, with over 90% of the world’s injury deaths occurring in low-and middle-income countries (LMIC) [1,2]. The financial demands associated with injuries pose particular difficulties for low-income families contributing to the ‘injury poverty trap’ [3-6].

Reliable epidemiological information is vital to guide the development of targeted injury prevention policies and strategies [7]. While some LMICs have established injury surveillance systems [8-13], most countries have difficulties implementing these due to limited resources. To assist member countries in these environments, the WHO has developed an *Injury Surveillance Guideline* advocating the collection of a minimum dataset [14].

Injury data from less resourced Pacific Island Countries and Territories has primarily relied on statistics in reports from government and non-government agencies responsible for health, law enforcement, transport, and social issues. Given the inadequacy of these data to inform robust national injury prevention efforts in Fiji, a prospective trauma registry was piloted and established in trauma admitting hospitals in Viti Levu, Fiji as part of the Traffic Related Injury in the Pacific (TRIP) Project [15]. The aim of this paper is to draw on this Fiji Injury Surveillance in Hospitals (FISH) system to describe the epidemiology of all injuries and to explore differences by socio-demographic characteristics (including ethnicity) and mechanisms of injury.

## Methods

This cross-sectional study used an adapted version of the WHO Injury Surveillance Guideline [14] to systematically collect information oninjury deaths and hospitalisations over a 12 month period (1 October 2005 to 30 September 2006) in all twelve trauma-admitting hospitals in Viti Levu, Fiji. At the 2007 census, approximately 70% of Fiji’s resident population of 837,271 people lived in Viti Levu [16]. The injury surveillance form captured data from medical records including demographic information, injury details (place of occurrence, activity, cause, intent, nature of the principal injury, length of stay, status at discharge) and recorded information on the likely influence of alcohol and kava. Kava is a mildly narcotic traditional brew made from the root of the *Piper methysticum* plant [17], widely consumed by all ethnic groups in Fiji.

Data collection was carried out by research assistants and hospital nurses located at the surveillance hospitals. Quality assurance measures were implemented to ensure cases met the inclusion criteria, and data collection and coding was complete and accurate [15].

All data management and analysis was conducted using Microsoft Excel Version 12.1.7 statistical software and Epi Info Version 3.3.2. Fatal and hospitalised injury incidence rates were calculated using denominator data from the 2007 census [16].

Ethical approval for the study was obtained from the Fiji National Research Ethics Committee and the University of Auckland Ethics Committee.

## Results

During the 12-month injury surveillance period, 2233 cases of injury were identified by the FISH system, 66 of whom were excluded from this analysis as they were transfers from hospitals outside of Viti Levu. The 2167 individuals who died or were admitted to hospital as a result of injury corresponded to an annual incidence of 333 per 100,000 population. Two thirds of those injured (69%) were male, more than half (56%) were indigenous Fijians. People aged 15 to 29 years and 30 to 44 years accounted for 35% and 23% of those injured, respectively.

### Injury admissions

During the review period, 2059 people were admitted to Viti Levu hospitals as a result of injury, 65 of whom were excluded as these were transfers from hospitals outside the study region. The remaining 1994 injury admissions corresponded to a crude annual rate of 306 per 100,000 (Table 1). Of these, 71 died in hospital (case fatality rate of 3.6%). People aged 15–29 years accounted for the highest admission rate (369 per 100,000). Males had twice the rate of admission of females. Unintentional injuries accounted for three-quarters of all injury admissions. The median length of hospital stay was 3.0 days (range 0 to 161 days).

**Table 1:** Injury admissions to hospital by gender, age group, ethnicity, and mechanism in Viti Levu, Fiji, October 2005-September 2006

**Variable**	**Overall § n (%)**	Overall rate* (95% CI)	**Fijian n (%)**	**Fijian rate (95% CI)**	**Indian, n (%)**	**Indian rate (95% CI)**
**Total**	1994	306.5 (293.0, 319.9)	1133	320.2 (301.5, 338.8)	756	290.8 (270.0, 311.5)
**Gender**						
Male	1373(68.9)	415.6 (393.6, 437.6)	808 (71.3)	452.3 (421.1, 483.5)	494 (65.3)	372.0 (339.2, 404.8)
Female	621 (31.1)	193.9 (178.7, 209.2)	325 (28.7)	185.4 (165.3, 205.6)	262 (34.7)	206.0 (181.0, 230.9)
**Age group**						
0 – 14 years	451 (2.6)	246.8 (224.0, 269.5)	311 (27.4)	274.8 (244.3, 305.4)	123 (16.3)	207.3 (170.6, 243.9)
15 – 29 years	694 (34.8)	368.6 (341.2, 396.0)	413 (36.5)	408.4 (369.0, 447.8)	247 (32.7)	321.4 (281.3, 361.5)
30 – 44 years	442 (22.2)	321.1 (291.2, 351.0)	259 (22.8)	361.3 (317.3, 405.3)	159 (21.0)	274.4 (231.7, 317.0)
≥ 45years	407(20.4)	286.8 (258.9, 314.6)	150 (13.2)	220.9 (185.5, 256.2)	227 (30.0)	344.7 (299.8, 389.5)
**Mechanism of injury**						
Fall	602 (30.2)	92.5 (85.1, 99.9)	309 (27.3)	87.3 (77.6, 97.0)	246 (32.5)	94.6 (82.8, 106.4)
Hit by person or object	466 (23.4)	71.6 (65.1, 78.1)	334 (29.5)	94.4 (84.3, 104.5)	108 (14.3)	41.5 (33.7, 49.4)
Road traffic injury	353(17.7)	54.3 (48.6, 59.9)	170 (15.0)	48.0 (40.8, 55.3)	171 (22.6)	65.8 (55.9, 75.6)
Cutting or piercing	228 (11.4)	35.0 (30.5, 39.6)	167 (14.7)	47.2 (40.0, 54.3)	50 (6.6)	19.2 (13.9, 24.6)
Poisoning	169 (8.5)	26.0 (22.1, 29.9)	48 (4.2)	13.6 (9.7, 17.4)	117 (15.5)	45 (36.8, 53.2)
Fire/heat/electricity	109 (5.5)	16.8 (13.6, 19.9)	62 (5.5)	17.5 (13.2, 21.9)	45 (6.0)	17.3 (12.3, 22.4)
Choking or hanging	17 (0.9)	2.6 (1.4, 3.9)	10 (0.9)	2.8 (1.1, 4.6)	6 (0.8)	2.3 (0.5, 4.2)
Sexual assault	9 (0.5)	1.4 (0.5, 2.3)	-	-	-	-
Near drowning	-	-	-	-	-	-
Other	18 (0.9)	2.8 (1.5, 4.0)	13 (1.1)	3.7 (1.7, 5.7)	-	-
Unknown	19 (1.0)	2.9 (1.6, 4.2)	13 (1.1)	3.7 (1.7, 5.7)	5 (0.7)	1.9 (0.2, 3.6)
**Intent**						
Unintentional	1493 (74.9)	229.5 (217.8, 241.1)	844 (74.5)	238.5 (222.4, 254.6)	568 (75.1)	218.5 (200.5, 236.4)
Self-inflicted injury	144 (7.2)	22.1 (18.5, 25.7)	30 (2.6)	8.5 (5.4, 11.5)	109 (14.4)	41.9 (34.1, 49.8)
Interpersonal violence	306 (15.3)	47 (41.8, 52.3)	23 (20.4)	65.3 (56.9, 73.7)	58 (7.7)	22.3 (16.6, 28.1)
Undetermined	51 (2.5)	7.8 (5.7, 10.0)	28 (2.5)	7.9 (5.0, 10.8)	21 (2.8)	8.1 (4.6, 11.5)
**Outcome**						
Discharged	1923 (96.4)	295.6 (282.3, 308.8)	1105 (97.5)	312.2 (293.8, 330.6)	715 (94.6)	275.0 (254.8, 295.1)
Died while admitted	71 (3.6)	10.9 (8.4, 13.5)	28 (2.5)	7.9 (5.0, 10.8)	41 (5.4)	15.98 (10.9, 20.6)
**Length of stay (days)**						
Total	13,515		7463		5371	
Mean	6.8		6.6		7.1	
Median	3 (0–161)		3 (0–107)		3 (0–161)	

Source:Fiji Bureau of Statistics 2007 census data [16]; * Rate per 100,000; **§ ‘**Other’ ethnic group is included in the overall count and rate calculation; - Cells with values less than 5 have been omitted.

The leading cause of injury admission was falls (30%) followed by ‘hit by a person or object’ (23%) and road traffic injury (18%), (Table 1). Among children less than 15 years of age and people aged 45 years and older, the leading cause of injury was falls. The commonest mechanism of injury in the 15 to 44 age group was being ‘hit by a person or object’.

Overall, indigenous Fijians had higher admission rates than Indians (Table 1). However, indigenous Fijians admission rates for males, and those aged <15, 15–29 and 30–44 years were higher than the corresponding Indian sub-groups; in contrast, the rate for indigenous Fijians aged greater than or equal to 45 years was lower than that among Indians.

The males aged 15–29 years in both ethnic groups (indigenous Fijians 619 per 100,000 and Indians 443 per 100,000) had the highest incidence rates, more than double the female rates. The same pattern was also observed in indigenous Fijians in the 30–44 years age group where the male rate was three times that of the indigenous Fijians female rate, (544 per 100,000 cf. 182 per 100,000).

Compared with Indians, indigenous Fijians had higher rates of admission for interpersonal violence. However, Indians had almost five times the rate of admission for self-inflicted injury. With respect to mechanism, indigenous Fijians had double the Indian rates of injury admission due to being hit by a person or object, and for cuts. In contrast, the rate of poisoning among Indians was three times higher than that among indigenous Fijians.

The principal injuries among those admitted to hospital were most commonly identified as fractures (41%) and open wounds (20%). Leisure or play (38%), travelling (19%) and being ‘in a conflict situation’ (18%) were common activities at the time of injury. Almost half the hospitalised injuries (47%) occurred in the home environment, and 21% occurred on the road.

Alcohol and kava use prior to the injury was documented in 12% and 3% of injury admissions, respectively, and the use of both substances together was documented in 21 cases. In 12% of cases, the use of alcohol was not recorded.

### Injury deaths

There were 246 injury deaths identified in the FISH database, two of whom were excluded because they were transfers from hospitals outside of Viti Levu. Of the remaining 244 fatalities (crude annual rate of 37.5 per 100,000), most (71%) occurred before admission (Table 2). Nearly two thirds (64%) of injury deaths were among people aged 15 to 44 years, and unintentional events accounted for 57% of all injury deaths.

**Table 2:** Injury deaths by gender, age group, ethnicity, and mechanism in Viti Levu, Fiji, October 2005-September 2006

**Variable**	**Overall § n (%)**	Overall rate* (95% CI)	**Fijian n (%)**	**Fijian rate (95% CI)**	**Indian n (%)**	**Indian rate (95% CI)**
**Total**	244	37.5 (32.8, 42.2)	98	27.7 (22.2, 33.2)	129	50 (41.1, 58.2)
**Gender**						
Male	156 (63.9)	47.2 (39.8, 54.6)	64 (65.3)	35.8 (27.0, 44.6)	80 (62.0)	60 (47.0, 73.4)
Female	88 (36.1)	27.5 (21.7, 33.2)	34 (34.7)	19.4 (12.9, 25.9)	49 (38.0)	39 (27.7, 49.3)
**Age group**						
0 – 14 years	28 (11.5)	15.3 (9.6, 21)	24 (24.5)	21.2 (12.7, 29.7)	-	-
15 – 29 years	95 (38.9)	50.5 (40.3, 60.6)	30 (30.6)	29.7 (19.0, 40.3)	60 (46.5)	78 (58.3, 97.8)
30 – 44 years	61 (25.0)	44.3 (33.2, 55.4)	28 (28.6)	39.1 (24.6, 53.5)	27 (20.9)	47 (29.0, 64.2)
≥ 45years	60 (24.6)	42.3 (31.6, 53.0)	16 (16.3)	23.6 (12.0, 35.1)	38 (29.5)	58 (39.4, 76.0)
**Mechanism of injury**						
Road traffic injury	70 (28.7)	10.8 (8.3, 13.3)	33 (33.7)	9.3 (6.1, 12.5)	35 (27.1)	13 (9.0, 17.9)
Choking or hanging	61 (25.0)	9.4 (7, 11.7)	13 (13.3)	3.7 (1.7, 5.7)	45 (34.9)	17 (12.3, 22.4)
Drowning	28 (11.5)	4.3 (2.7, 5.9)	18 (18.4)	5.1 (2.7, 7.4)	-	-
Fire/heat/electricity	22 (9.0)	3.4 (2.0, 4.8)	-	-	17 (13.2)	7 (3.4, 9.6)
Poisoning	17 (7.0)	2.6 (1.4, 3.9 )	-	-	13 (10.1)	5 (2.3 - 7.7)
Fall	16 (6.6)	2.5 (1.3, 3.7 )	9 (9.2)	2.5 (0.9, 4.2)	6 (4.7)	2 (0.5, 4.2)
Hit by person or object	12 (4.9)	1.8 (0.8, 2.9 )	8 (8.2)	2.3 (0.7, 3.8)	-	-
Cutting or piercing	11 (4.5)	1.7 (0.7, 2.7)	5 (5.1)	1.4 (0.2, 2.7)	5 (3.9)	2 (0.2, 3.6)
Other	7 (2.9)	1.1 (0.4, 1.9)	6 (6.1)	1.7 (0.3, 3.1)	-	-
**Intent**						
Unintentional	138 (56.5)	21.2 (17.7, 24.7)	70 (71.4)	19.8 (15.1, 24.4)	57 (44.2)	21.9 (16.2, 27.6)
Self-inflicted injury	78 (32.0)	12.0 (9.3, 14.6)	9 (9.2)	2.5 (0.9, 4.2)	64 (49.6)	24.6 (18.6, 30.6)
Interpersonal violence	19 (7.8)	2.9 (1.6, 4.2)	13 (13.3)	3.7 (1.7, 5.7)	6 (4.7)	2.3 (0.5, 4.2)
Undetermined	9 (3.7)	1.4 (0.5, 2.3)	6 (6.1)	1.7 (0.3, 3.1)	-	-
**Timing of death**						
Prior to admission	173 (70.9)	26.6 (22.6, 30.6)	70 (71.4)	19.8 (15.1, 24.4)	88 (68.2)	34 (26.7, 40.9)
During admission	71 (29.1)	10.9 (8.4, 13.5)	28 (28.6)	7.9 (5.0, 10.8)	41 (31.8)	16 (10.9, 20.6)

Source: Fiji Bureau of Statistics 2007 census data [16]; * Rate per 100,000; **§ ‘**Other’ ethnic group is included in the overall count and rate calculation; - Cells with values less than 5 have been omitted.

The leading cause of injury-related deaths was road traffic injury (29%) followed by ‘choking or hanging’ (25%) and drowning (12%). Road traffic injury was the leading cause of injury death in all age groups except the 15–29 age groups where ‘choking or hanging’ was more common.

In contrast to admission rates, Indians had double the fatality rate of indigenous Fijians both in males and females (Table 2). Similar to admissions, Indians had higher mortality rates due to self-inflicted injury, but mortality rate due to interpersonal violence was close to that registered among indigenous Fijians.

‘Choking or hanging’ (35%), road traffic injury (27%), and ‘fire, heat or electricity’ related injuries (13%) were the commonest causes of injury deaths among Indians. Road traffic injury (34%), drowning (18%) and ‘choking or hanging’ (13%) were the commonest in indigenous Fijians. ‘Choking or hanging’ and ‘fire, heat or electricity’-related deaths were higher among Indians compared to indigenous Fijians.

Injury deaths were most commonly attributed to asphyxia (36%) and head injury (20%). The context of injury deaths were most often defined as a ‘conflict situation’ (32%), or while travelling (26%). Close to half of the injury deaths (48%) occurred at home.

Of the 244 injury deaths, 13% were considered to have involved alcohol use, (17% males cf. 6% females). Alcohol use was recorded as ‘unknown’ among 28 females and 55 males. Kava use was suspected or confirmed in 2% of deaths.

## Discussion

This analysis of fatal and hospitalized injury in Viti Levu, Fiji, indicates that substantial burden of acute injury involving males and people in the economically-productive ages of 15 to 44 years. The most common locations of injury were the home and road. Nearly three-quarters of injury events were unintentional with road traffic injuries and falls comprising the leading causes of injury death and admissions respectively. ‘Hanging or choking’ and assault were the leading causes of intentional injury death and hospitalisation. Most injury deaths occurred before admission. Indigenous Fijians had higher rates of injury admission, more than half of which were due to being ‘hit by a person or object’ and falls. People of Indian ethnicity had higher rates of fatality, especially from suicide. While alcohol use was implicated in injury deaths and hospital admissions, the contribution of substances, in general, is underestimated in the absence of systematic enquiry and objective measures.

The population-based Fiji Injury Surveillance in Hospitals (FISH) system has demonstrated that it is feasible to obtain a comprehensive policy-relevant profile of injury using a simple data collection form in low-resourced Pacific nations. The hospitals involved in this study were located throughout the main island, and covered urban and rural environments.

The findings must also be interpreted in light of several limitations. While the pilot phase of this study attempted to ensure mechanisms of particular relevance for this context were captured, the efficiency of the simple data collection form also required compromises with regard to the specificity of information. For example, some broad categories of injuries (such as fire, heat or electricity) limited our ability to look at each group separately.

The principal injury captured by the FISH system was the one deemed the most serious in terms of threat to life. However, misclassification of this variable is likely in the absence of a formal injury severity coding system. As the study was restricted to deaths and hospital admissions, injuries that are less severe but could result in disability were not identified.

The findings in relation to the predominance of males and road traffic injuries are consistent with trends seen in other developing countries [10,12,13,18]. While data on the specific types of road users was not available in this study, data from other LMICs report pedestrians, bicyclists, and motorcyclists as the most vulnerable groups [12,19-21].

More than two thirds of the injury deaths identified in this study, occurred before arrival in hospital – a high proportion relative to other developing countries [22,23]. Mock et al. in their analysis of mortality trends in three nations with different economic levels showed that pre-hospital deaths declined with increased economic level [22]. The majority of pre-hospital care in Fiji is provided by ambulance services aligned to regional hospitals. The findings indicate the need to review the effectiveness of emergency response services including training of personnel, and transfer times to definitive care.

Ethnic-specific differences by intent showed consistent patterns for injury deaths and admissions. Indigenous Fijians had higher rates of interpersonal violence while Indians had higher rates of self-inflicted harm. The latter finding is consistent with findings from previous research in Fiji [24], India [25,26], and other LMICs.

The suspicion of alcohol use prior to injury, particularly among males aged 15–44, is consistent with the international literature [27]. However, both alcohol and kava use as contributing factors in this study are likely to have been under-estimated in a country where blood alcohol testing is not routinely done unless requested by Police, and kava use is common among people, often in combination with alcohol [28].

The absence of an objective measure for injury severity was a limitation of the FISH system. While scoring methods applied in high-income countries may be difficult to implement due to available resources and training, the use or adaptation of a score such as the Kampala Trauma Score [29] could be a useful adjunct to data collected on trauma patients in Fiji. This would provide the opportunity to risk adjust when considering changes in the profiles of injury over time, the impact of interventions, and the quality of pre-hospital and in-patient trauma care.

Previous publications using injury surveillance systems established in Thailand [30], Ethiopia [12], Jamaica [8], South Africa [31], and Nicaragua [13] have identified some limitations in data collection similar to our study. Particular issues identified include: challenges to assuring data quality, inadequate human resources, incomplete and inaccurate coding, and a lack of a surveillance culture. Collectively, these studies also reveal the importance of integrating data collection into patient registration systems as key to the success of surveillance systems.

Limited resources can challenge the establishment of robust public health surveillance systems in low resourced countries [32]. However, attention to characteristics that support the reliability and sustainability of a surveillance system [33] would provide the opportunity to monitor future trends in injury in Fiji, and evaluate the impact of preventive interventions. Mitchell et al. identify three priority areas that are especially pertinent to injury surveillance systems [34]. These include (1) ‘data quality’- to ensure the information collected is complete, representative, valid and reliable; (2) the operational capacity - to ensure that the surveillance system has a clear purpose and objectives, case definitions, data collection process while also being simple, flexible and responsive to change; and (3) ‘practical capability’-to ensure the accessibility, acceptability and usefulness of data, incorporating the necessary resource allocation, communication support, and training.

As a component of a wider research project, the FISH system was designed to generate a population-based profile of acute injury events in Viti Levu over 12 months. The project demonstrated that a simple yet efficient, standardized data collection system could be effectively implemented in Viti Levu. The experience gained with FISH is timely given the proposed national accident and injury surveillance system identified in the Fiji Non-Communicable Disease Strategic Plan (2010 – 14) [35]. Of relevance, Fiji has recently launched a national Health Information policy [36] modeled on the Health Metrics Network (HMN) framework [37]. The core objective of this policy is to provide timely and quality information to support health service planning. The findings of the present study alongside these national initiatives provide the impetus to design, implement and evaluate a comprehensive injury control strategy in Fiji. The strategies required include giving priority attention to preventing road traffic injury, falls, intentional injuries and alcohol-related injuries, as well as attention to improving outcomes across the injury continuum, particularly including effective pre-hospital care. The study also identifies the need to examine the context-specific risk and protective factors that can address the burden of injuries in less-resourced Pacific Island nations.

## Conclusions

Injury is an important public health problem that disproportionately affects young males in Viti Levu, Fiji. A high proportion of injury-related deaths occur prior to hospital presentation. This study highlights road traffic injury, falls, intentional injuries and alcohol-related injuries as key areas requiring priority attention to reduce the burden of potentially life-threatening injuries. With local initiatives to develop a national injury prevention strategy and enhance health information systems, there is a critical opportunity to address injury control as a major public health program in countries with limited resources in the Pacific.

## Competing interests

The authors declare that they have no competing interests.

## Authors' contributions

IW helped design the study, was one of two research managers supervising the conduct of the study, and wrote the initial draft of the manuscript. BKafoa helped design the study, was one of two research managers supervising the conduct of the study, and contributed to the interpretation of the study findings. BKool contributed to the analysis and interpretation of the study results, and assisted with preparation of the manuscript. JH assisted with the statistical analysis of the study results and with the development of the manuscript. EM is a co-principal investigator of the project; he contributed to the interpretation of the study findings, and critically revising the draft manuscript. SA is the project director and a co-principal investigator obtaining funding for the study, contributed to the design and supervised the implementation and analysis of the study, interpretation of findings, and critically revising the draft manuscript for important intellectual content. All authors read and approved the final manuscript.

## Pre-publication history

The pre-publication history for this paper can be accessed here:

http://www.biomedcentral.com/1471-2458/12/1074/prepub
